# Evaluation of antiplasmodial activity of medicinal plants from North Indian Buchpora and South Indian Eastern Ghats

**DOI:** 10.1186/s12936-015-0564-z

**Published:** 2015-02-07

**Authors:** Naveen K Kaushik, Asokan Bagavan, Abdul A Rahuman, Abdul A Zahir, Chinnaperumal Kamaraj, Gandhi Elango, Chidambaram Jayaseelan, Arivarasan V Kirthi, Thirunavukkarasu Santhoshkumar, Sampath Marimuthu, Govindasamy Rajakumar, Santosh K Tiwari, Dinkar Sahal

**Affiliations:** Malaria Research Group, International Centre for Genetic Engineering and Biotechnology, Aruna Asaf Ali Marg, New Delhi, 110067 India; Unit of Nanotechnology and Bioactive Natural Products, Department of Zoology, C. Abdul Hakeem College, Melvisharam 632509, Vellore District, Tamil Nadu India; Department of Bioscience & Biotechnology, Banasthali University, P.O. Banasthali Vidyapith, Rajasthan, 304 022 India; Present address: Department of Genetics, Maharshi Dayanand University, Rohtak, Haryana 124001 India

## Abstract

**Background:**

Development of resistance against the frontline anti-malarial drugs has created an alarming situation, which requires intensive drug discovery to develop new, more effective, affordable and accessible anti-malarial agents.

**Methods:**

Inspired by their ethnobotanical reputation for being effective against febrile diseases, antiplasmodial potential of ethyl acetate extracts (EAE) and methanol extracts (ME) of 17 medicinal plants collected from the Eastern Ghats of South India and Buchpora, North India were explored against *Plasmodium falciparum in vitro* using the SYBR Green assay. The results were validated both by confirmation that the fall in fluorescence signal was not due to quenching effects mediated by phytochemical extracts and by Giemsa-stained microscopy.

**Results:**

Using EAE or ME, promising antiplasmodial activity (IC_50_*Pf*3D7 ≤ 20 μg/ml), was seen in *Aerva lanata* (Whole aerial parts-EAE), *Anisomeles malabarica* (Leaf-EAE)*, Anogeissus latifolia* (bark-EAE), *Cassia alata* (leaves-EAE), *Glycyrrhiza glabra* (root-EAE), *Juglans regia* (seed-ME), *Psidium guajava* (leaf-ME and EAE) and *Solanum xanthocarpum* (Whole aerial parts-EAE). EAEs from leaves of *Couroupita guianensis*, *Euphorbia hirta*, *Pergularia daemia, Tinospora cordifolia* and *Tridax procumbens* as also ME from *Ricinus communis* (leaf and seed) showed good antiplasmodial activity (*Pf* 3D7 IC_50_ 21 - 40 μg/ml). Moderate activity (*Pf* 3D7 IC_50_: 40–60 μg/mL) was shown by the leaf EAEs of *Cardiospermum halicacabum*, *Indigofera tinctoria* and *Ricinus communis* while the remaining extracts showed marginal (*Pf* 3D7 IC_50_ 60 to >100 μg/ml) activities. The promising extracts showed good resistance indices (0.41 – 1.4) against the chloroquine resistant INDO strain of *P. falciparum* and good selectivity indices (3 to > 22.2) when tested against the HeLa cell line.

**Conclusion:**

These results provide validity to the traditional medicinal usage of some of these plants and further make a case for activity-guided purification of new pharmacophores against malaria.

**Electronic supplementary material:**

The online version of this article (doi:10.1186/s12936-015-0564-z) contains supplementary material, which is available to authorized users.

## Background

In the absence of a credible vaccine and with emergence of resistance to almost all anti-malarial drugs, the dream of eradication of malaria appears to be a huge challenge. Caused by a protozoan parasite, malaria remains one of the dreaded diseases of the developing world, killing 367,000–755,000 people and causing disease in 124–283 million people annually [1]. The most severe manifestations of malaria are caused by *Plasmodium falciparum*. Even as malaria has been affecting both the economic and emotional aspects of mankind for a long time, the relief against malaria has been coming in the form of herbal treatments, such as cinchona bark and Qing Hao leaves, which gave quinine and artemisinin respectively. The quinoline-based quinine first and chloroquine later proved to be effective therapies against malaria till resistance against quinolines began to surface and spread to large parts of the world [2]. Against this scenario, artemisinin proved to be a smart, fast acting, potent drug against chloroquine-resistant malaria. However, artemisinin resistance in the form of delayed clearance of the parasite is now on the horizon[3] conjuring images of a world where mankind may be left with no effective drug against malaria. This calls for a rigorous search for novel anti-malarials.

One optimistic source for new affordable treatments against malaria lies in the use of traditional herbal remedies. Despite the recent successes in rational drug design and synthetic chemistry techniques by pharmaceutical companies, natural products and particularly medicinal plants have remained an important source of new drugs [4,5]. A definite virtue with medicinal plants is the rich ethnopharmacological history of traditional knowledge and usage associated with them. It is quite possible that their use as nutrients or spices may already be providing a significant degree of protection to people at large against malaria. However if the gist of traditional knowledge can be validated by scientific experiments, affordable and dependable cures can be found against the drug resistant dreaded forms of malaria. Further such exploratory endeavours can pave the path for identifying novel pharmacophores against malaria, which can be chemically synthesized and fine tuned as drugs of the future. With this perspective in mind, here is described the antiplasmodial potential of the extracts of 17 medicinal plants having the reputation of their usage against febrile diseases.

## Methods

### Methodology of collection of ethnomedical information

Recommendation of traditional healers and available literature were referred for selection and collection of medicinal plants. Information regarding the pharmacological usage of these plants is given in Additional file 1.

### Identification and collection of plant materials

**Figure 1:**
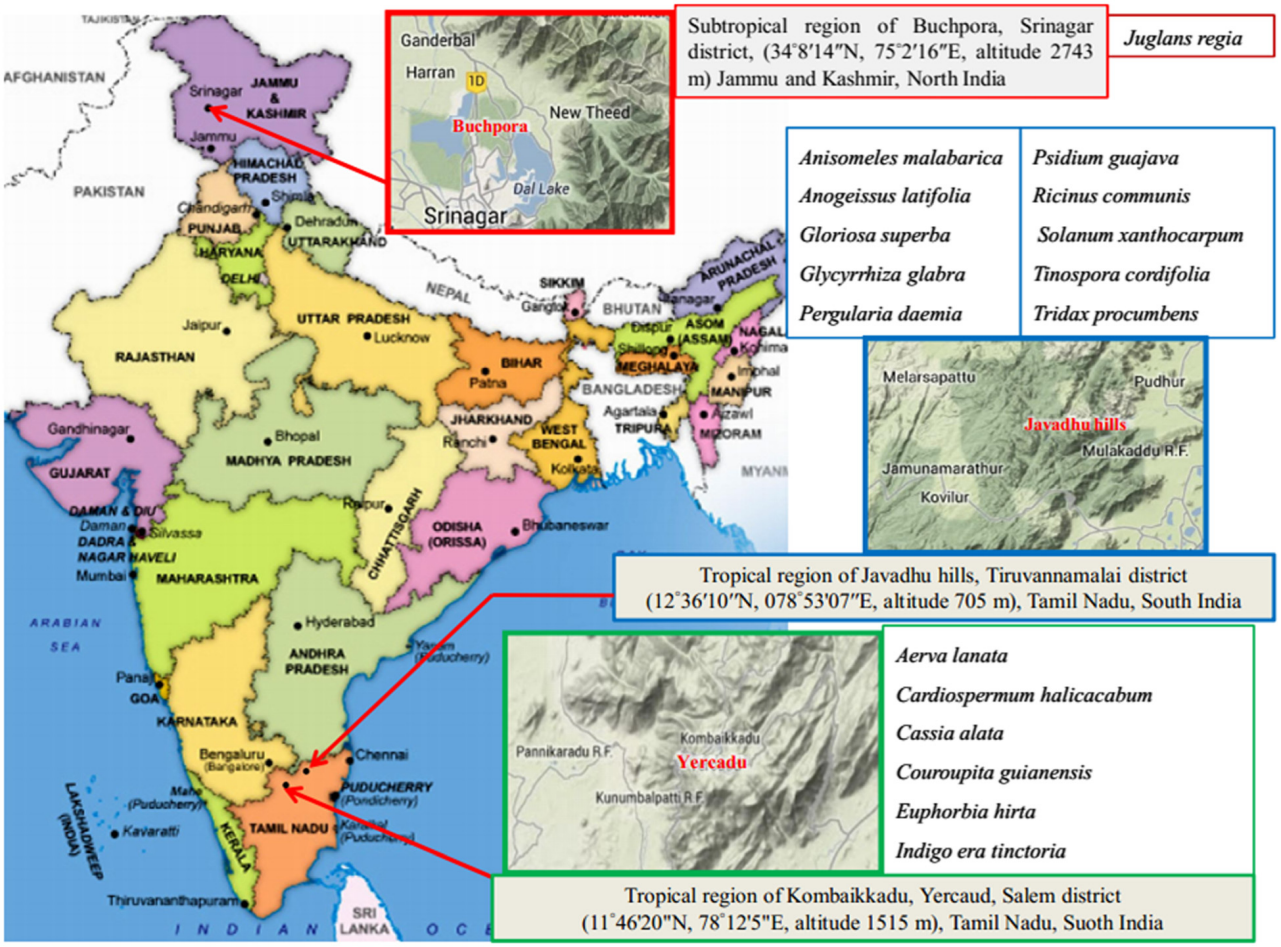
**Sites of collection of medicinal plants from North Indian Buchpora and South Indian Eastern Ghats.**

The seeds of *Juglans regia* (Juglandaceae) were collected from the Buchpora, Srinagar district, (34°8′14″N 75°2′16″E, altitude 2743 m) of Jammu and Kashmir, North India between October and November 2010 (Figure 1). The leaves of *Anisomeles malabarica* (Lamiaceae), *Psidium guajava* (Myrtaceae), *Tridax procumbens* (Asteraceae), leaves and seeds of *Ricinus communis* (Euphorbiaceae), and the flowers of *Gloriosa superba* (Liliaceae), *Pergularia daemia*, *Tinospora cordifolia*, bark of *Anogeissus latifolia*, root of *Glycyrrhiza glabra*, and whole aerial parts of *Solanum xanthocarpum* were collected from the tropical region of Javadhu Hills, Jamunamarathur, Tiruvannamalai district (12°36′10″N, 078°53′07″E, altitude 705 m), Tamil Nadu, South India (Figure 1). The leaves of *Cardiospermum halicacabum, Cassia alata*, *Couroupita guianensis*, *Euphorbia hirta*, *Indigofera tinctoria* and whole aerial parts of *Aerva lanata* were collected from the Eastern Ghats, Kombaikkadu, Yercaud, Salem district (11°46′20″N, 78°12′5″E, altitude 1,515 m), Tamil Nadu, South India. The taxonomic identifications of collected samples were made by Dr. C. Hema, Department of Botany, Arignar Anna Govt. Arts College for Women, Walajapet, Vellore, India, following which the voucher specimens were numbered and kept in laboratory for further reference. During raw material collection, sustainable harvesting was practiced in order to protect the habitat. For each medicinal plant, information about its vernacular name, the part used, preparation, administration and posology was obtained (Additional file 1).

### Preparation of crude plant extracts

The collected plants samples were air-dried for 7–30 days in the shade at the environmental temperatures (27°C–37°C) and the leaves (250 g), flowers (550 g), seeds (350 g) were powdered mechanically using a commercial electrical stainless steel blender and extracted with a) ethyl acetate (Qualigens) and b) methanol (Qualigens) in a Soxhlet apparatus (boiling point range 60–80°C) for 8 h. Extracts were concentrated on a rotary evaporator under a reduced pressure of 22–26 mm Hg at 45°C and the residues obtained were weighed and stored at 4°C.

### *In vitro* cultivation of *Plasmodium falciparum*

Chloroquine (CQ)-sensitive strain 3D7 and CQ-resistant strain INDO of *Plasmodium falciparum* were used for *in vitro* blood stage culture to test the antiplasmodial efficacy of different plant extracts. The culture was maintained at the Malaria Research Laboratory, International Centre for Genetic Engineering and Biotechnology, New Delhi, India. *Plasmodium falciparum* culture was maintained according to the method described by Trager and Jensen [6] with minor modifications, in fresh O + ve human erythrocytes suspended at 4% haematocrit in RPMI 1640 (Sigma) containing 0.2% sodium bicarbonate, 0.5% albumax I, 45 μg/L hypoxanthine, and 50 μg/L gentamicin and incubated at 37°C under a gas mixture of 5% O_2_, 5% CO_2_, and 90% N_2_. Every day, infected erythrocytes were transferred into fresh complete medium to propagate the culture.

### Dilutions of drugs and test samples

Stock solutions of plant extracts and artemisinin were prepared in dimethyl sulfoxide (DMSO) while CQ stock solution was in water (Milli-Q grade). All stocks were then diluted with culture medium to achieve the required concentrations (in all cases except CQ, the final solution contained 0.4% DMSO, which was found to be non-toxic to the parasite). Drugs and plant extracts were then placed in 96-well flat bottom tissue culture grade plates.

### *In vitro* antiplasmodial assays on *Plasmodium falciparum* in human red blood cell culture

The extracts of experimental plants were evaluated for their antiplasmodial activity against 3D7 and INDO strains of *P. falciparum*. For drug screening, SYBR green I-based fluorescence assay was set up as described [7]. Sorbitol synchronized parasites [8] (100 μl) were incubated under normal culture conditions at 2% haematocrit and 1% parasitaemia in the absence or presence of increasing concentrations of plant extracts. CQ and artemisinin were used as positive controls, while 0.4% (v/v) DMSO was used as the negative control. After 48 h of incubation, 100 μl of SYBR Green I lysis buffer was added to each well and mixed twice gently with multi-channel pipette and incubated in dark at 37°C for 1 h. Fluorescence was measured with a Victor fluorescence multi-well plate reader (PerkinElmer, Waltham, MA) with excitation and emission wavelength bands centered at 485 and 530 nm, respectively. The fluorescence counts were plotted against the drug concentration and the 50% inhibitory concentration (IC_50_) was determined by analysis of dose response curves. Results were validated microscopically by examination of Giemsa stained smears of extract treated parasite cultures. In view of the fluorescence basis of the SYBR Green assay, it was important to assess artefacts due to quenching effects caused by phytochemicals present in each of the plant extracts tested. To accomplish this, parasite cultures (2% haematocrit and 10% parasitaemia) were incubated with or without test extracts (100 μg/ml) for 1 hr at 37°C following which 100 μl of SYBR green lysis buffer was added and further incubated in dark at 37°C for 1 h. Fluorescence was measured with a Victor fluorescence multi-well plate reader with excitation and emission wavelength bands centered at 485 and 530 nm, respectively. Fluorescence of treated and untreated cultures was compared to detect quenching effects.

### Cytotoxic activity on HeLa cells using MTT assay

The cytotoxic effects of extracts on host cells were assessed by functional assay as described in ref [9] using HeLa cells cultured in RPMI containing 10% foetal bovine serum, 0.21% sodium bicarbonate (Sigma) and 50 μg/mL gentamicin (complete medium). Briefly, cells (10^4^ cells/200 μl/well) were seeded into 96 - well flat-bottom tissue culture plates in complete medium. Drug solutions were added after 24 h of seeding and incubated for 48 h in a humidified atmosphere at 37°C and 5% CO_2_. DMSO (as positive inhibitor) was added at 10% v/v. Twenty micro liters of a stock solution of MTT (5 mg/mL in 1X phosphate buffered saline) was added to each well, gently mixed and incubated for another 4 h. After spinning the plate at 1,500 RPM, 30°C for 5 min, supernatant was removed and 100 μl of DMSO (stop agent) was added. Formation of formazan was read on a microtiter plate reader (VersaMax Microplate Reader, Molecular Devices, USA) at 570 nm. The 50% cytotoxic concentration (TC_50_) of test samples was determined by analysis of dose–response curves. Therapeutic index was calculated as a ratio of TC_50_ HeLa /IC_50_ 3D7.

## Results and discussion

**Figure 2:**
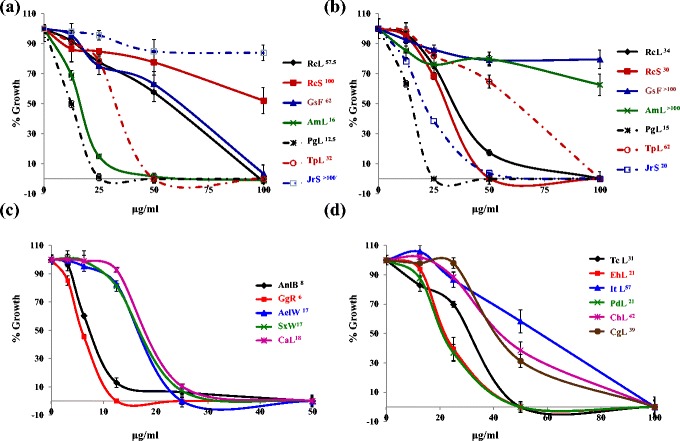
**Dose dependent growth inhibition curves of** ***Plasmodium falciparum*** **3D7 by plant extracts. (a)** ethyl acetate extracts and **(b)** methanol extracts of *Ricinus communis* leaf (RcL), *Ricinus communis* seed (RcS)*, Gloriosa superba* flower (GsF)*, Anisomeles malabarica* leaf (AmL)*, Psidium guajava* leaf (PgL)*, Tridax procumbens* leaf (TpL) and *Juglans regia* seed (JrS). **c)** ethyl acetate extracts of *Anogeissus latifolia* Bark (AnlB), *Glycyrrhiza glabra* Roots (GgR), *Aerva lanata* whole aerial part (AelW), *Solanum xanthocarpum* whole aerial part (SxW), *Cassia alata* leaves (CaL) and **d)** ethyl acetate extracts of *Tinospora cordifolia* leaves (TcL), *Euphorbia hirta* leaves (Eh), *Indigofera tirictoria* leaves (ItL), *Pergularia daemia* leaves (PdL), *Cardiospermum helicacabum* leaves (ChL) and *Couroupita guianensis* leaves (CgL). Superscript numbers in label index refer to IC_50_ (μg/mL).

Indigenous healthcare systems have always played a vital role in the management of community health and discovery of novel chemotherapeutic agents. Medicinal plants which offer a wide diversity of medicinal properties have proved to be a boon for malaria therapy as two of the most important anti-malarial drugs, namely quinine and artemisinin, have their origins in the medicinal plants *Cinchona officinalis* and *Artemisia annua,* respectively. Ethnomedicinal plants could be attractive start materials as they are wide spread and also a large population relies on them for their curative effects. In the present study, 17 medicinal plants known for their traditional medicinal usage (Additional file 1) and pharmacological activities (Additional file 2) were evaluated for (a) their antiplasmodial activity against CQ-sensitive *P. falciparum* 3D7 and CQ-resistant *P. falciparum* INDO strains and (b) their toxicity against HeLa cell line (Table 1). Among the seventeen plants studied, seven (*Aerva lanata*, *Anisomeles malabarica, Anogeissus latifolia, Couroupita guianensis, Indigofera tinctoria, Juglans regia* and *Solanum xanthocarpum*) have been tested for their antiplasmodial activity against both the 3D7 and INDO strains for the first time. As shown in Figure 2 and Table 1, five of these seven plants showed promising (*Pf*3D7 IC_50_ 6 μg/ml to 20 μg/ml) antiplasmodial activity. Interestingly four of these plant extracts showed greater potency against the CQ-resistant INDO strain than against the CQ-sensitive 3D7 strain. Further the selectivity indices (HeLa cells *versus P. falciparum*) for the promising extracts ranged from 3 to >22 (Table 1).

**Table 1:** **Antiplasmodial activity, Cytotoxicity and selectivity of methanol (M) and ethyl acetate (EA) extracts of selected medicinal plants**

**Sr No.**	**Name of the plants**	**Parts and Solvent of Extraction M-Methanol EA-Ethyl acetate**	**Yields (%)**	***P. falciparum*** **IC**_**50**_ **(μg/mL)**		**Cytotoxicity (TC**_**50**_ **μg/mL) against HeLa cell line**
				**3D7**	**INDO**	
1	*Aerva lanata*	Whole aerial EA	15.4	17	8 (0.47)*	77 (9.63)^#^
2	*Anisomeles malabarica*	Leaf M		>100	-	-
		Leaf EA		16	16 (1)	48 (3)^#^
3	*Anogeissus latifolia*	Bark EA	12.5	8	4.5(0.56)	>100 (>22)
4	*Cardiospermum halicacabum*	Leaves EA	10.2	42	-	-
5	*Cassia alata*	Leaves EA	14.1	18	20 (1.11)	100 (5)
6	*Couroupita guianensis*	Leaves EA	7.6	39	-	-
7	*Euphorbia hirta*	Leaves EA	9.6	21	-	-
8	*Gloriosa superba*	Flower M		>100	-	-
		Flower EA		62	-	-
9	*Glycyrrhiza glabra*	Root EA	13.2	6	4.5 (0.75)	22 (4.89)
10	*Indigofera tinctoria*	Leaves EA	8.5	57	-	-
11	*Juglans regia*	Seed M		20	12.5 (0.625)	>100 (>8)
		Seed EA		>100	-	-
12	*Pergularia daemia*	Leaves EA	12.9	21	-	-
13	*Psidium guajava*	Leaf M		15	9 (0.6)	68 (4.6)
		Leaf EA		12.5	18 (1.4)	82 (6.6)
14	*Ricinus communis*	Leaf M		34	-	-
		Leaf EA		57.5	-	-
		Seed M		30	-	-
		Seed EA		100	-	-
15	*Solanum xanthocarpum*	Whole aerial EA	11.6	17	7 (0.41 )	75 (10.7)
16	*Tinospora cordifolia*	Leaves EA	6.8	31	-	-
17	*Tridax procumbens*	Leaf M		62	-	-
		Leaf EA		32	-	-
	Chloroquine			0.021	0.258 (12.28)	>200
	Artemisinin			0.0045	0.0045 (1)	>200

(−) not tested, *values in parentheses represent resistance indices (IC_50_ INDO/IC_50_ 3D7), ^#^values in parentheses represent selectivity indices (TC_50_ HeLa/IC_50_INDO).

For the remaining ten plants, which have been studied for antiplasmodial activity by previous investigators, experiments were performed to find which part of plant and solvent of extraction could contribute phytochemicals with superior antiplasmodial activity. In this context, leaf methanol and ethyl acetate extracts of *Psidium guajava* were found to show promising antiplasmodial activity (IC_50_*Pf*3D7: 15 & 12.5 μg/ml respectively) (Table 1, Figure 2). Earlier Nundkumar and Ojewole [10] have reported promising activity of aqueous stem-bark extract of *Psidium guajava* against CQ-sensitive *P. falciparum* D10 strain (IC_50_: 20 μg/ml). This suggests that *Psidium guajava* leaves could be a better source for antiplasmodial molecules than stem-bark since leaves are renewable while girding for stem bark can cause severe injury to the tree.

Methanol extracts of seed and leaf of *Ricinus communis* (IC_50_*Pf*3D7: 30 & 34 μg/ml respectively) showed good antiplasmodial activity. However the corresponding ethyl acetate extracts (IC50 *Pf*3D7 > 71μg/ml) showed moderate to poor antiplasmodial activity (Table 1, Figure 2). Clarkson *et al.* [11] also reported similar moderate activity (IC_50_*P. falciparum* D10: 27.5 μg/ml) in *R. communis* leaves DCM-methanol extract whereas they reported promising activity in its stem dichloromethane/methanol extract (IC_50_*P. falciparum* D10: 8 μg/ml).

Leaf ethyl acetate extract of *Tridax procumbens* showed good activity (IC_50_ 3D7: 32 μg/ml) whereas the corresponding methanol extract showed moderate antiplasmodial activity (IC_50_ 3D7: 62 μg/ml) (Table 1, Figure 2). However Appiah-Opong *et al.* [12] reported poor antiplasmodial activity in whole plant extracts (water, ethanol, chloroform and ethyl acetate) (IC_50_: 140–430 μg/ml). This suggests that leaves should be preferred over the whole plant and extraction should be made in ethyl acetate for enriching antiplasmodial molecules.

Ethyl acetate flower extract of *Gloriosa superba* showed moderate (IC_50_ 3D7: 62 μg/ml) antiplasmodial activity whereas corresponding methanol extract was found to be inactive up to 100 μg/ml (Table 1 and Figure 2). Bagavan *et al.* [13] have reported that ethyl acetate and methanol extracts of the leaves of *Gloriosa superba* have good antiplasmodial effect against CQ resistant strain of *P. falciparum* INDO (IC_50_: 30–40 μg/ml) which suggests that leaves of *Gloriosa superba* may be preferred over its flowers as a source of antiplasmodial molecules.

*Cardiospermum halicacabum* also known as the balloon plant is a climbing plant widely distributed in tropical and subtropical Africa and Asia. As shown in Figure 2 and Table 1, the leaves ethyl acetate extract of this plant was found to have moderate antiplasmodial activity (IC_50_*Pf*3D7: 42 μg/mL). Clarkson *et al.* [11] reported that the dichloromethane / methanol (1:1) whole balloon plant extract showed good *in vitro* antiplasmodial activity (*P. falciparum* D10 IC_50_: 20 μg/mL) whereas Waako *et al.* [14] extracted its shoots with different solvents and reported moderate to poor activity in ethyl acetate (*P. falciparum* D10 IC_50_: 28 μg/mL) and methanol extracts (*P. falciparum* D10 IC_50_: 62 μg/mL). This comparative study suggests that the stem of *Cardiospermum halicacabum* may have more potent antiplasmodial molecules which makes shoots ethyl acetate extract more potent than leaves ethyl acetate extract.

*Cassia alata* is an important medicinal as well as an ornamental flowering plant of subfamily Caesalpinioideae. The results described here indicate promising antiplasmodial activity in the ethyl acetate extract of *C. alata* against both CQ sensitive 3D7 (IC_50_: 18 μg/mL) and CQ resistant INDO (IC_50_: 20 μg/mL) strains with low toxicity to HeLa cells (TC_50_: 100 μg/mL). However, Zirihi *et al.* [15] found no antiplasmodial activity up to 50 μg/mL in leaf ethanol extract of *Cassia alata* whereas Kayembe *et al.* [16] reported promising antiplasmodial activity (IC_50_: 12.5 μg/mL) in the seed ethanol extract of *Cassia alata*. This suggests that ethyl acetate is a better solvent for extraction of promising molecules from leaves compared to ethanol whereas ethanol is equally good in their extraction from seeds.

*Euphorbia hirta* is a pantropical weed, native to India. It is a hairy herb that grows in open grasslands, roadsides and pathways. It is widely used as a medicinal herb in most places it grows (Additional file 1). In the present study, good antiplasmodial activity (IC_50_*Pf*3D7: 21 μg/mL) has been found in leaves ethyl acetate extract of *Euphorbia hirta*. However, Tona *et al.* [17] who studied the ethanol, petroleum ether and isoamyl alcohol extracts of *Euphorbia hirta* whole plant have reported promising antiplasmodial activity (IC_50_: 2.4, 1.2 and 2.6 μg/mL respectively). This indicates the importance of solvents and choice of plant parts towards enriching promising metabolites.

*Glycyrrhiza glabra* (liquorice) is a herb belonging to the pea and bean family, and is cultivated for its underground stems that are used to flavour confectionery. In the present study, the roots of this plant were found to have promising anti-plasmodial activity against both 3D7 and INDO strains (IC_50_: 6 & 4.5 μg/mL). Prior reports indicate that methanol extract [18] of its aerial parts exhibits poor antiplasmodial activity against CQ sensitive 3D7 (IC_50_: >64 μg/mL) and good activity against the CQ resistant K1(IC_50_: 17.5 μg/mL) strain of *P. falciparum*. The results reported here suggest that roots may be better source for antiplasmodial molecules than the aerial part of the plant.

*Pergularia daemia* is a hispid, perennial vine of Apocynaceae family, with an extensive range in the Old World tropics and subtropics. It has been used traditionally to treat a number of ailments (Additional file 1). Kantamreddi and Wright [19] studied the leaves methanol extract of *Pergularia daemia* against 3D7 and K1 strains of *P. falciparum* and indicated it to be poorly antiplasmodial (IC_50_: 203.8 and 244.1 μg/mL, respectively). However in the present study, extraction of leaves with ethyl acetate resulted in about 10-fold potentiation of antiplasmodial activity (*Pf*3D7IC_50_: 21 μg/mL) reiterating the crucial role of solvents in extraction of metabolites that hold promise.

*Tinospora cordifolia* is used in the Indian Ayurvedic system of medicine for the treatment of jaundice, diabetes, and rheumatoid arthritis, and is also used as an immunostimulant. Simonsen *et al.* [20] reported that the stem ethanol extract of *Tinospora cordifolia* is poorly antiplasmodial (IC_50_*Pf*3D7: 62 μg/mL). However, Tran *et al.* [21] have reported that the stem methanol and methanol : water (1:1) extracts with IC_50_ 6.1 and 3.2 μg/mL, respectively against FCR-3 strain of *P. falciparum* possessed promising antiplasmodial activity. In the present study it was found that ethyl acetate leaf extract of *Tinospora cordifolia* is moderately antiplasmodial (*Pf*3D7IC_50_: 31 μg/mL) against 3D7 strain of *P. falciparum.*

Promising extracts (*Pf*3D7IC_50_: < 20 μg/mL) of *Aerva lanata*, *Anisomeles malabarica, Anogeissus latifolia, Cassia alata, Glycyrrhiza glabra, Juglans regia, Psidium guajava* and *Solanum xanthocarpum* on further analysis against chloroquine resistant INDO strain also showed good resistance indices (0.41 – 1.4) suggesting that they may be equally effective against both chloroquine-sensitive and resistant-strains of *Plasmodium*. Further the selectivity indices of 3 to > 22.2 observed with some of the plant extracts studied by us suggest that they exhibit considerable selectivity against the malaria parasite over the mammalian HeLa cell line (Table 1).

**Figure 3:**
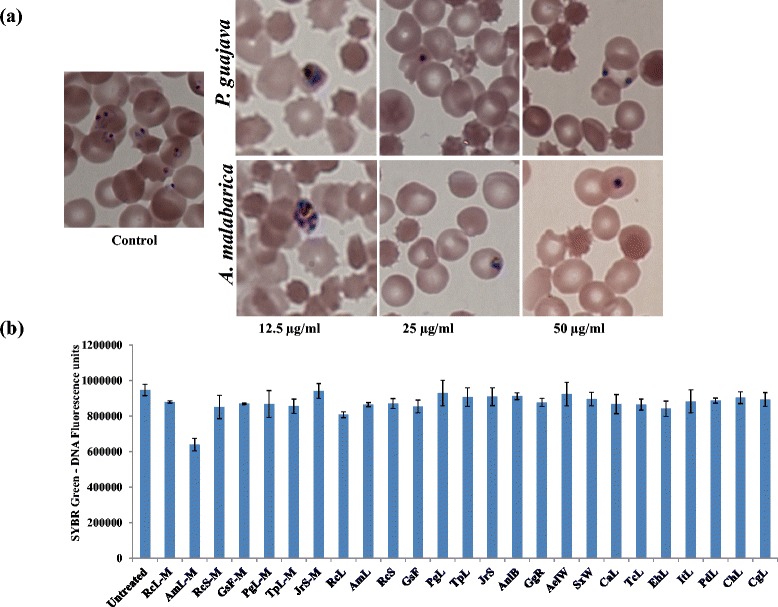
**Validation of SYBR Green results by Microscopy and estimation of quenching of fluorescence by plant extracts. (a)** Micrographs of synchronized ring stage parasite cultures treated with ethyl acetate extract of *Anisomeles malabarica* leaf and *Psidium guajava* leaf after 48 h. Note that at 12.5 μg/ml, the parasitemia is decreased and the parasite is arrested at trophozoite stage in case of *Pg* while the arrest in case of *Am* is at early schizont stage. **(b)** Examination of fluorescence quenching effects in plant extracts: Untreated and extract treated (100 μg/ml) parasite cultures (10% parasitemia) were subjected to SYBR Green fluorescence intensity measurements. The nearly identical intensities across untreated control and extract treated test samples indicate that the test extracts showed no significant quenching of SYBR green 1-DNA fluorescence.

The results of SYBR Green assay described above were further validated by microscopy and experimental estimation of SYBR Green fluorescence in parasitized red blood cells in presence *vs* absence of the plant extracts studied here (Figure 3). Microscopic evaluation of ethyl acetate extracts of *Anisomeles malabarica* and *Psidium guajava* showed dose dependent inhibition of parasite growth (Figure 3a). Further none of twenty five extracts studied by us showed any significant quenching effects at 100 μg/ml (Figure 3b) providing validity to the SYBR Green assay based results described above.

These results indicate a possible explanation of the traditional use of some of these medicinal plants against malaria or malaria like conditions. These results are significant since they report for the first time broad spectrum antiplasmodial activities in the extracts of *Aerva. lanata*, *Anisomeles malabarica, Anogeissus latifolia, Cassia alata, Couroupita guianensis, Glycyrrhiza glabra, Indigofera tinctoria Juglans regia, Psidium guajava* and *Solanum xanthocarpum.* Further both plant parts and preferred solvents were identified that provide extracts of high antiplasmodial potency. This paves the paths for (a) standardized plant extracts based therapy against malaria and (b) for antiplasmodial activity guided isolation of new pharmacophores and their subsequent development towards phytochemical based novel drugs against malaria.

## Additional files

Additional file 1:
**Medicinal properties of selected plant species.**
Additional file 2:
**Ethnobotanical exploration and biological activity of selected medicinal plants.**
