# Supplementary material for: Evaluation of antiplasmodial activity of medicinal plants from North Indian Buchpora and South Indian Eastern Ghats
Source: Malar J. 2015 Feb 7;14:65. doi: 10.1186/s12936-015-0564-z (PMC4340493; doi:10.1186/s12936-015-0564-z)
Supplement: Additional file 1: — Medicinal properties of selected plant species. [file 12936_2015_564_MOESM1_ESM.docx]

**Additional file 1 Table:** **Medicinal properties of selected plant species.**

| **S.No** | **Name of the plants / Family / Vernacular name / Parts used / Growth habit** | **Plant Distribution** | **Common medicinal usage** |
| --- | --- | --- | --- |
| 1 | *Aerva lanata* (L.) A. L. Juss. ex Schultes. / Amaranthaceae / Perumpoolai / Whole aerial parts / Herb | It is prostrate herbaceous weed, common throughout the hotter AND tropical region of Asia Africa and Australia. | The root is crushed, squeezed and juice is given to cure liver congestion, jaundice, biliousness and dyspepsia. Decoction of the whole plant to cure pneumonia, typhoid and other prolonged fevers. Leaves are macerated and few drops are given orally to the asthmatic child at the time of wheezing. |
| 2 | *Anisomeles malabarica* / Lamiaceae / Irattaippeymarutti/ Shrub | Native to tropical and subtropical Australia, Malaysia, Southeast Asia and Mauritius | Used to treat amentia, anorexia, fevers, swelling, and rheumatism. |
| 3 | *Anogeissus latifolia* (Roxb. ex DC.) Wall. ex Guill. & Perr. / Combretaceae / Vellainagai / Bark / Tree | It is commonly found in dry as well as moist deciduous forests and grows on a variety of soils. These trees constitute one of the largest forest coverages in India. | Ethnobotanically, the bark has been reported to be used in the treatment of various disorders like skin diseases, snake and scorpion bite, mouth sores, stomach diseases, colic, cough and diarrhoea. |
| 4 | *Cardiospermum halicacabum* Linn. / Sapindaceae / Mudukottan / Leaves / Climber | It is an herbaceous twiny climber which is found throughout the plains of India | Plant extract is taken orally against constipation, and for ﬂatulence. It has been used in Ayurveda and folk medicine and the seeds were used as a tonic for fevers. |
| 5 | *Cassia alata* Linn. / Caesalpiniaceae / Simayakatti / Leaves / Shrub | It is wild growing shrub distributed in tropics. The plant is native to Southeast Asia, Fiji, Northern Australia, Africa and Latin America. | The leaves paste is used to treat ringworm. Root powder is mixed with lime juice and applied topically on the affected places to treat skin diseases. |
| 6 | *Couroupita guianensis* Aubl. / Lecythidaceae / Naagalingam / Leaves / Tree | *C. guianensis* is commonly called “Ayahuma” or “Cannonball tree” distributed in the tropical regions of Northern South America and Southern Caribbean and also India. | Traditionally, the leaves are used as antiseptic and toothache. The juice made from the leaves is used to cure skin diseases. The tree parts are used to cure colds and stomach aches. The juice made from the leaves is used to cure skin diseases, and the Shamans of South America have even used the tree parts for treating malaria. |
| 7 | *Euphorbia hirta* L., / Euphorbiaceae / Amman pachharisi / Leaves / Herb | It is annual herb can get up to 80 cm long with a solid, hairy stem that produced abundant white latex. It is a rhizomatous herb distributed in southern Western Ghats of India and northern east coast of Tamil Nadu. | The 10–20 g leaves are crushed into paste, mixed in whey and given orally in diarrhea. The fresh latex is applied to warts and the treatment needs to be repeated 2–3 times a day. Whole plant is decocted and taken internally and paste is also applied on affected area in the treatment of athlete’s foot. The fresh leaves juice (one teaspoon 3-5 times daily) is given in cough and asthma. |
| 8 | *Gloriosa superba /* Liliaceae / Nabhikkodi / Climber | Native to Africa and Asia, but grown worldwide as an ornamental plant | The tuberous roots are useful in treatment of gout, skin diseases, inflammation, ulcers, bleeding piles, leprosy, and snakebites. The Ulanga people of Tanzania drink the juice of this plant for treatment of malaria. |
| 9 | *Glycyrrhiza glabra* Linn / Fabaceae / Athimaduram / Root / Herb | Licorice is a traditional medicinal herb growing in the various parts of the World. It is a hardy herb or under shrub, erects and grows to about 2 m high. The roots are long, cylindrical, thick and multi-branched | The roots and rhizomes of licorice (Glycyrrhiza) species have long been used worldwide as an herbal medicine and natural sweetener. The root is a traditional medicine used mainly for the treatment of peptic ulcer, hepatitis C, and pulmonary and skin diseases. This plant traditional uses from Iran to treat gastric ulcers, hepatic disorders and malaria. |
| 10 | *Indigofera tinctoria*Linn. / Fabaceae / Avuri / Leaves / Shrub | It is an herb, bears the common name true indigo. It has been naturalized to tropical and temperate Asia, as well as parts of Africa | The plant was one of the original sources of indigo dye. The root powder is given orally to cure dropsy. Root is useful for promoting the growth of hairs. The juice expressed from leaves is useful in the treatment of hydrophobia. The herb is widely used in Indian and Chinese systems of medicine. |
| 11 | *Juglans regia /* Juglandaceae / Akhrot / Tree | Native to the mountain ranges of Central Asia. | Used for diabetes, cardioprotective, dietary source of essential fatty acids, vitamins and minerals. The seed coat is used in Calabria folk medicine to treat malaria.. |
| 12 | *Pergularia daemia* (Forssk.) Chiov / Asclepiadaceae / Vaeliparuththi / Leaves / Herb | *P. daemia* is a perennial herb growing widely along the road sides of India. | It has been used in folk medicine for the treatment of liver disorders. The plant is useful as anthelmintic, laxative, anti-pyretic and expectorant, and is also used in infan tile diarrhoea. |
| 13 | *Psidium guajava /* Myrtaceae / Koyya / Tree | Evergreen shrub native to Mexico, Caribbean, Central and South America | Fruit is edible. Used for diabetes, women's healthcare, anti-inflammatory, caries, wounds, gastrointestinal and respiratory disturbances, hypertension, pain relief and reducing fever. The leaf and fruit are used for treatment of malaria by traditional medicinal practitioners of Chalna, Khulna district, Bangladesh. |
| 14 | *Ricinus communis* / Euphorbiaceae / Aamanakku / Shrub | Indigenous to the south eastern Mediterranean Basin, Eastern Africa and India | Body ointments, improving hair growth and texture, eye ointment, laxative, arthritic diseases. The entire plant decoction is traditionally used for the treatment of malaria in Ngazidja island (Comoros). |
| 15 | *Solanum xanthocarpum* Burm.f. / Solanaceae / Kandankatthiri / Whole aerial parts / Erect | Commonly known as “Yellow Berried Nightshade”,it is a prickly diffuse bright green perennial herb, woody at the base, 2–3 m height found in India, mostly in dry places as a weed on roadsides. | Dried or fresh fruits are kept in fire and the smoke is inhaled through mouth to treat toothache. Ayurveda, it is better, appetiser, laxative, anthelmintic, stomachic, and useful inbronchitis, asthma, fever, lumbago, pains, piles (specially bleeding piles), thirst, urinary and heart. |
| 16 | *Tinospora cordifolia* Miers / Menispermaceae / Seenil / Leaves / Climber | *T. cordifolia* is commonly known as “Guduchi” or “Giloe” is a large, extensively spreading, glabrous, perennial deciduous shrub and widely distributed throughout the plains of India. | Leaves are smeared with oil or butter, warmed and tied over fractured bone to cure early. Stem juice with sweet oil is administered for elephantiasis, and the juice is given to malaria patients. It has been used as antimalarial property in tribal area of Rajasthan, India The decoction of stem bark is recommended orally twice a day for a week used as remedy of jaundice. Fruit- boiled in milk and drunk for 10-15 days treated tuberculosis. |
| 17 | *Tridax procumbens /* Asteraceae/ Vettukkaaya-thalai / Herb | The plant bears daisy like yellow-centered white or yellow flowers with three-toothed ray florets native to tropical Americas | Used for wound healing, anticoagulant, antifungal, insect repellent, diarrhea, dysentery, skin diseases, hepatoprotective. The entire plant is used by indigenous people in Guatemala region for the treatment of protozoal infections including malaria. |
