# Supplementary material for: Evaluation of antiplasmodial activity of medicinal plants from North Indian Buchpora and South Indian Eastern Ghats
Source: Malar J. 2015 Feb 7;14:65. doi: 10.1186/s12936-015-0564-z (PMC4340493; doi:10.1186/s12936-015-0564-z)
Supplement: Additional file 2: — Ethnobotanical exploration and biological activity of selected medicinal plants. [file 12936_2015_564_MOESM2_ESM.docx]

**Additional File 2 Table: Ethnobotanical exploration and biological activity of selected medicinal plants**

| **S. No** | **Species name** | **Biological activities** |
| --- | --- | --- |
| 1 | *A. lanata* | Antidiabetic [[1](#_ENREF_1)], antioxidant[[2](#_ENREF_2)], diuretic, anti-inflammatory, hypoglycemic, anti-diabetic, antiparasitic, antimicrobial, hepoprotective, anti-urolithiasis, antiasthmatic, antifertility, hypolipidemic, Immunomodulatory and antitumor[[3](#_ENREF_3)]. |
| *2* | *A. malabarica* | Anticancer[[4](#_ENREF_4)], anthelmintic[[5](#_ENREF_5)], antileishmanial [[6](#_ENREF_6)], larvicidal [[7](#_ENREF_7)], antiepileptic [[8](#_ENREF_8)]. |
| *3* | *A. latifolia* | Antiulcer and antimicrobial activity [[9](#_ENREF_9)]. Antioxidant activity[[10](#_ENREF_10)] and hepatoprotective activity[[11](#_ENREF_11)]. |
| *4* | *C. halicacabum* | Antioxidant and anti-inﬂammatory[[12](#_ENREF_12)], antimalarial activity [[13](#_ENREF_13)] |
| *5* | *C. alata* | Antimicrobial and antifungal[[14](#_ENREF_14)], antiplasmodial[[15](#_ENREF_15)], antitumor [[16](#_ENREF_16)],) antidiabetic [[17](#_ENREF_17)] and anthelmintic [[18](#_ENREF_18)] |
| *6* | *C. guianensis* | Anti-inflammatory[[19](#_ENREF_19)], antioxidant and anticancer activities [[20](#_ENREF_20)] and antimicrobial [[21](#_ENREF_21)] |
| *7* | *E. hirta* | Antioxidant [[22](#_ENREF_22)], immunomodulatory [[23](#_ENREF_23)], antiviral [[24](#_ENREF_24)], antiplasmodial [[25](#_ENREF_25)] and antidiabetic activity[[26](#_ENREF_26)] |
| *8* | *G. superba* | Antileishmanial [[27](#_ENREF_27)], Antihaemolytic and snake venom neutralizing [[28](#_ENREF_28)], antiosteoclastogenesis [[29](#_ENREF_29)], larvicidal[[30](#_ENREF_30)], antiplasmodial [[31](#_ENREF_31)]. |
| *9* | *G. glabra* | Antiinﬂammatory, antiviral, antimicrobial, antioxidative, anticancer activities, immunomodulatory, hepatoprotective and cardioprotective activities [[32](#_ENREF_32)] and antifilarial [[33](#_ENREF_33)] |
| *10* | *I. tinctoria* | Hepatoprotective [[34](#_ENREF_34)], anticancer [[35](#_ENREF_35)] |
| *11* | *J. regia* | Antidiabitic[[36](#_ENREF_36)], antileishmanial [[37](#_ENREF_37)], mollusicidal [[38](#_ENREF_38)], hepatoprotective [[39](#_ENREF_39)], antioxidant[[40](#_ENREF_40)], antimicrobial[[41](#_ENREF_41)], anticancerous [[42](#_ENREF_42)], |
| *12* | *P. daemia* | Anti-inflammation, antidiabetes, antiplasmodial, antiasthmatic [[43](#_ENREF_43)] and hepatoprotective activity [[44](#_ENREF_44)]. |
| *13* | *P. guajava* | Anti-diarrhoeal [[45](#_ENREF_45)], antimicrobial [[46](#_ENREF_46)], antiplasmodial [[47](#_ENREF_47)], antitussive[[48](#_ENREF_48)], antigenotoxic [[49](#_ENREF_49)], anticancerous [[50](#_ENREF_50)] |
| *14* | *R. communis* | Antimicrobial [[51](#_ENREF_51)], Antinociceptive[[52](#_ENREF_52)], larvicidal [[53](#_ENREF_53)], anti-inflammatory [[54](#_ENREF_54)], Antiplasmodial [[55](#_ENREF_55)]. |
| *15* | *S. xanthocarpum* | Antioxidant [[56](#_ENREF_56)], and antifungal activity [[57](#_ENREF_57)] |
| *16* | *T. cordifolia* | Antimicrobial, Antioxidant activity [[58](#_ENREF_58)], antidiabetic [[59](#_ENREF_59)], hypoglycemic activity [[60](#_ENREF_60)] and antimicrobial activity [[61](#_ENREF_61)]. |
| *17* | *T. procumbens* | Hepatoprotective [[62](#_ENREF_62)], vasorelaxant [[63](#_ENREF_63)], antiarthritic[[64](#_ENREF_64)], anticancerous [[65](#_ENREF_65)], antimicrobial[[66](#_ENREF_66)], antiplasmodial [[67](#_ENREF_67)], larvicidal[[68](#_ENREF_68)], antileishmanial [[69](#_ENREF_69)], Immunomodulatory [[70](#_ENREF_70)]. |

**References**

1. Agrawal R, Sethiya NK, Mishra SH: **Antidiabetic activity of alkaloids of Aerva lanata roots on streptozotocin-nicotinamide induced type-II diabetes in rats.** *Pharm Biol* 2013, **51:**635-642.

2. Kumar G, Karthik L, Rao KV: **Phytochemical composition and in vitro antioxidant activity of aqueous extract of Aerva lanata (L.) Juss. ex Schult. Stem (Amaranthaceae).** *Asian Pac J Trop Med* 2013, **6:**180-187.

3. Goyal M, Pareek A, Nagori BP, Sasmal D: **Aerva lanata: A review on phytochemistry and pharmacological aspects.** *Pharmacogn Rev* 2011, **5:**195-198.

4. Preethy CP, Alshatwi AA, Gunasekaran M, Akbarsha MA: **Analysis of the Cytotoxic Potential of Anisomelic Acid Isolated from Anisomeles malabarica.** *Sci Pharm* 2013, **81:**559-566.

5. Kamaraj C, Rahuman AA, Elango G, Bagavan A, Zahir AA: **Anthelmintic activity of botanical extracts against sheep gastrointestinal nematodes, Haemonchus contortus.** *Parasitol Res* 2011, **109:**37-45.

6. Zahir AA, Rahuman AA, Pakrashi S, Ghosh D, Bagavan A, Kamaraj C, Elango G, Chatterjee M: **Evaluation of antileishmanial activity of South Indian medicinal plants against Leishmania donovani.** *Exp Parasitol* 2012, **132:**180-184.

7. Elango G, Rahuman AA, Kamaraj C, Bagavan A, Zahir AA: **Screening for feeding deterrent activity of herbal extracts against the larvae of malaria vector Anopheles subpictus Grassi.** *Parasitol Res* 2011, **109:**715-726.

8. Choudhary N, Bijjem KR, Kalia AN: **Antiepileptic potential of flavonoids fraction from the leaves of Anisomeles malabarica.** *J Ethnopharmacol* 2011, **135:**238-242.

9. Govindarajan R, Vijayakumar M, Singh M, Rao Ch V, Shirwaikar A, Rawat AK, Pushpangadan P: **Antiulcer and antimicrobial activity of Anogeissus latifolia.** *J Ethnopharmacol* 2006, **106:**57-61.

10. Govindarajan R, Vijayakumar M, Rao CV, Shirwaikar A, Rawat AK, Mehrotra S, Pushpangadan P: **Antioxidant potential of Anogeissus latifolia.** *Biol Pharm Bull* 2004, **27:**1266-1269.

11. Pradeep HA, Khan S, Ravikumar K, Ahmed MF, Rao MS, Kiranmai M, Reddy DS, Ahamed SR, Ibrahim M: **Hepatoprotective evaluation of Anogeissus latifolia: in vitro and in vivo studies.** *World J Gastroenterol* 2009, **15:**4816-4822.

12. Huang MH, Huang SS, Wang BS, Wu CH, Sheu MJ, Hou WC, Lin SS, Huang GJ: **Antioxidant and anti-inflammatory properties of Cardiospermum halicacabum and its reference compounds ex vivo and in vivo.** *J Ethnopharmacol* 2011, **133:**743-750.

13. Waako PJ, Gumede B, Smith P, Folb PI: **The in vitro and in vivo antimalarial activity of Cardiospermum halicacabum L. and Momordica foetida Schumch. Et Thonn.** *J Ethnopharmacol* 2005, **99:**137-143.

14. Ibrahim D, Osman H: **Antimicrobial activity of Cassia alata from Malaysia.** *J Ethnopharmacol* 1995, **45:**151-156.

15. Kayembe J, Taba K, Ntumba K, Tshiongo M, Kazadi T: **In vitro anti-malarial activity of 20 quinones isolated from four plants used by traditional healers in the Democratic Republic of Congo.** *J Med Plant Res* 2010, **4:**991-994.

16. Olarte EI, Herrera AA, Villasenor IM, Jacinto SD: **In vitro antitumor properties of an isolate from leaves of Cassia alata L.** *Asian Pac J Cancer Prev* 2013, **14:**3191-3196.

17. Varghese GK, Bose LV, Habtemariam S: **Antidiabetic components of Cassia alata leaves: identification through alpha-glucosidase inhibition studies.** *Pharm Biol* 2013, **51:**345-349.

18. Kundu S, Roy S, Lyndem LM: **Cassia alata L: potential role as anthelmintic agent against Hymenolepis diminuta.** *Parasitol Res* 2012, **111:**1187-1192.

19. Pinheiro MM, Bessa SO, Fingolo CE, Kuster RM, Matheus ME, Menezes FS, Fernandes PD: **Antinociceptive activity of fractions from Couroupita guianensis Aubl. leaves.** *J Ethnopharmacol* 2010, **127:**407-413.

20. Premanathan M, Radhakrishnan S, Kulangiappar K, Singaravelu G, Thirumalaiarasu V, Sivakumar T, Kathiresan K: **Antioxidant & anticancer activities of isatin (1H-indole-2,3-dione), isolated from the flowers of Couroupita guianensis Aubl.** *Indian J Med Res* 2012, **136:**822-826.

21. Al-Dhabi NA, Balachandran C, Raj MK, Duraipandiyan V, Muthukumar C, Ignacimuthu S, Khan IA, Rajput VS: **Antimicrobial, antimycobacterial and antibiofilm properties of Couroupita guianensis Aubl. fruit extract.** *BMC Complement Altern Med* 2012, **12:**242.

22. Basma AA, Zakaria Z, Latha LY, Sasidharan S: **Antioxidant activity and phytochemical screening of the methanol extracts of Euphorbia hirta L.** *Asian Pac J Trop Med* 2011, **4:**386-390.

23. Ramesh KV, Padmavathi K: **Assessment of Immunomodulatory Activity of Euphorbia hirta L.** *Indian J Pharm Sci* 2010, **72:**621-625.

24. Gyuris A, Szlavik L, Minarovits J, Vasas A, Molnar J, Hohmann J: **Antiviral activities of extracts of Euphorbia hirta L. against HIV-1, HIV-2 and SIVmac251.** *In Vivo* 2009, **23:**429-432.

25. Tona L, Cimanga RK, Mesia K, Musuamba CT, De Bruyne T, Apers S, Hernans N, Van Miert S, Pieters L, Totte J, Vlietinck AJ: **In vitro antiplasmodial activity of extracts and fractions from seven medicinal plants used in the Democratic Republic of Congo.** *J Ethnopharmacol* 2004, **93:**27-32.

26. Kumar S, Malhotra R, Kumar D: **Antidiabetic and Free Radicals Scavenging Potential of Euphorbia hirta Flower Extract.** *Indian J Pharm Sci* 2010, **72:**533-537.

27. Zahir AA, Rahuman AA, Bagavan A, Geetha K, Kamaraj C, Elango G: **Evaluation of medicinal plant extracts and isolated compound epicatechin from Ricinus communis against Paramphistomum cervi.** *Parasitol Res* 2012, **111:**1629-1635.

28. Kumarapppan C, Jaswanth A, Kumarasunderi K: **Antihaemolytic and snake venom neutralizing effect of some Indian medicinal plants.** *Asian Pac J Trop Med* 2011, **4:**743-747.

29. Reuter S, Gupta SC, Phromnoi K, Aggarwal BB: **Thiocolchicoside suppresses osteoclastogenesis induced by RANKL and cancer cells through inhibition of inflammatory pathways: a new use for an old drug.** *Br J Pharmacol* 2012, **165:**2127-2139.

30. Bagavan A, Kamaraj C, Elango G, Abduz Zahir A, Abdul Rahuman A: **Adulticidal and larvicidal efficacy of some medicinal plant extracts against tick, fluke and mosquitoes.** *Vet Parasitol* 2009, **166:**286-292.

31. Bagavan A, Rahuman AA, Kaushik NK, Sahal D: **In vitro antimalarial activity of medicinal plant extracts against Plasmodium falciparum.** *Parasitol Res* 2011, **108:**15-22.

32. Asl MN, Hosseinzadeh H: **Review of pharmacological effects of Glycyrrhiza sp. and its bioactive compounds.** *Phytother Res* 2008, **22:**709-724.

33. Kalani K, Kushwaha V, Verma R, Murthy PK, Srivastava SK: **Glycyrrhetinic acid and its analogs: a new class of antifilarial agents.** *Bioorg Med Chem Lett* 2013, **23:**2566-2570.

34. Singh B, Saxena AK, Chandan BK, Bhardwaj V, Gupta VN, Suri OP, Handa SS: **Hepatoprotective activity of indigtone--a bioactive fraction from Indigofera tinctoria Linn.** *Phytother Res* 2001, **15:**294-297.

35. Han R: **Highlight on the studies of anticancer drugs derived from plants in China.** *Stem Cells* 1994, **12:**53-63.

36. Hosseini S, Huseini HF, Larijani B, Mohammad K, Najmizadeh A, Nourijelyani K, Jamshidi L: **The hypoglycemic effect of Juglans regia leaves aqueous extract in diabetic patients: A first human trial.** *Daru* 2014, **22:**19.

37. Serakta M, Djerrou Z, Mansour-Djaalab H, Kahlouche-Riachi F, Hamimed S, Trifa W, Belkhiri A, Edikra N, Hamdi Pacha Y: **Antileishmanial activity of some plants growing in Algeria: Juglans regia, Lawsonia inermis and Salvia officinalis.** *Afr J Tradit Complement Altern Med* 2013, **10:**427-430.

38. Jiang JM, Yang L, Fei SM, Mo KL, Sun QX: **[Mollusicidal effects of some species of plants in hilly and mountainous areas].** *Zhongguo Xue Xi Chong Bing Fang Zhi Za Zhi* 2013, **25:**255-258.

39. Eidi A, Moghadam JZ, Mortazavi P, Rezazadeh S, Olamafar S: **Hepatoprotective effects of Juglans regia extract against CCl4-induced oxidative damage in rats.** *Pharm Biol* 2013, **51:**558-565.

40. Chen N, Yang H, Sun Y, Niu J, Liu S: **Purification and identification of antioxidant peptides from walnut (Juglans regia L.) protein hydrolysates.** *Peptides* 2012, **38:**344-349.

41. Zakavi F, Golpasand Hagh L, Daraeighadikolaei A, Farajzadeh Sheikh A, Daraeighadikolaei A, Leilavi Shooshtari Z: **Antibacterial Effect of Juglans Regia Bark against Oral Pathologic Bacteria.** *Int J Dent* 2013, **2013:**854765.

42. Hafeez BB, Zhong W, Mustafa A, Fischer JW, Witkowsky O, Verma AK: **Plumbagin inhibits prostate cancer development in TRAMP mice via targeting PKCepsilon, Stat3 and neuroendocrine markers.** *Carcinogenesis* 2012, **33:**2586-2592.

43. Bhaskar VH, Balakrishnan N: **Pharmacognostic studies on Pergularia daemia roots.** *Pharm Biol* 2010, **48:**427-432.

44. Sureshkumar SV, Mishra SH: **Hepatoprotective effect of extracts from Pergularia daemia Forsk.** *J Ethnopharmacol* 2006, **107:**164-168.

45. Tona L, Kambu K, Mesia K, Cimanga K, Apers S, De Bruyne T, Pieters L, Totte J, Vlietinck AJ: **Biological screening of traditional preparations from some medicinal plants used as antidiarrhoeal in Kinshasa, Congo.** *Phytomedicine* 1999, **6:**59-66.

46. Chah KF, Eze CA, Emuelosi CE, Esimone CO: **Antibacterial and wound healing properties of methanolic extracts of some Nigerian medicinal plants.** *J Ethnopharmacol* 2006, **104:**164-167.

47. Nundkumar N, Ojewole JA: **Studies on the antiplasmodial properties of some South African medicinal plants used as antimalarial remedies in Zulu folk medicine.** *Methods Find Exp Clin Pharmacol* 2002, **24:**397-401.

48. Jaiarj P, Khoohaswan P, Wongkrajang Y, Peungvicha P, Suriyawong P, Saraya ML, Ruangsomboon O: **Anticough and antimicrobial activities of Psidium guajava Linn. leaf extract.** *J Ethnopharmacol* 1999, **67:**203-212.

49. Grover IS, Bala S: **Studies on antimutagenic effects of guava (Psidium guajava) in Salmonella typhimurium.** *Mutat Res* 1993, **300:**1-3.

50. Manosroi J, Dhumtanom P, Manosroi A: **Anti-proliferative activity of essential oil extracted from Thai medicinal plants on KB and P388 cell lines.** *Cancer Lett* 2006, **235:**114-120.

51. Naz R, Bano A, Ilyas N: **Antimicrobial potential of Ricinus communis leaf extracts in different solvents against pathogenic bacterial and fungal strains.** *Asian Pac J Trop Biomed* 2012, **2:**944-947.

52. Taur DJ, Waghmare MG, Bandal RS, Patil RY: **Antinociceptive activity of Ricinus communis L. leaves.** *Asian Pac J Trop Biomed* 2011, **1:**139-141.

53. Elimam AM, Elmalik KH, Ali FS: **Larvicidal, adult emergence inhibition and oviposition deterrent effects of foliage extract from Ricinus communis L. against Anopheles arabiensis and Culex quinquefasciatus in Sudan.** *Trop Biomed* 2009, **26:**130-139.

54. Lomash V, Parihar SK, Jain NK, Katiyar AK: **Effect of Solanum nigrum and Ricinus communis extracts on histamine and carrageenan-induced inflammation in the chicken skin.** *Cell Mol Biol (Noisy-le-grand)* 2010, **56 Suppl:**OL1239-1251.

55. Clarkson C, Maharaj VJ, Crouch NR, Grace OM, Pillay P, Matsabisa MG, Bhagwandin N, Smith PJ, Folb PI: **In vitro antiplasmodial activity of medicinal plants native to or naturalised in South Africa.** *J Ethnopharmacol* 2004, **92:**177-191.

56. Kumar S, Sharma UK, Sharma AK, Pandey AK: **Protective efficacy of Solanum xanthocarpum root extracts against free radical damage: phytochemical analysis and antioxidant effect.** *Cell Mol Biol (Noisy-le-grand)* 2012, **58:**174-181.

57. Dabur R, Singh H, Chhillar AK, Ali M, Sharma GL: **Antifungal potential of Indian medicinal plants.** *Fitoterapia* 2004, **75:**389-391.

58. Pushp P, Sharma N, Joseph GS, Singh RP: **Antioxidant activity and detection of (-)epicatechin in the methanolic extract of stem of Tinospora cordifolia.** *J Food Sci Technol* 2013, **50:**567-572.

59. Sangeetha MK, Priya CD, Vasanthi HR: **Anti-diabetic property of Tinospora cordifolia and its active compound is mediated through the expression of Glut-4 in L6 myotubes.** *Phytomedicine* 2013, **20:**246-248.

60. Patel MB, Mishra S: **Hypoglycemic activity of alkaloidal fraction of Tinospora cordifolia.** *Phytomedicine* 2011, **18:**1045-1052.

61. Samy RP: **Antimicrobial activity of some medicinal plants from India.** *Fitoterapia* 2005, **76:**697-699.

62. Adetutu A, Olorunnisola OS: **Hepatoprotective Potential of Some Local Medicinal Plants against 2-Acetylaminoflourene-Induced Damage in Rat.** *J Toxicol* 2013, **2013:**272097.

63. Salahdeen HM, Idowu GO, Murtala BA: **Endothelium-dependent and independent vasorelaxant effects of aqueous extract of Tridax procumbens Lin. leaf in rat aortic rings.** *Indian J Exp Biol* 2012, **50:**883-888.

64. Petchi RR, Vijaya C, Parasuraman S: **Anti-arthritic activity of ethanolic extract of Tridax procumbens (Linn.) in Sprague Dawley rats.** *Pharmacognosy Res* 2013, **5:**113-117.

65. Manjamalai A, Kumar MJ, Grace VM: **Essential oil of Tridax procumbens L induces apoptosis and suppresses angiogenesis and lung metastasis of the B16F-10 cell line in C57BL/6 mice.** *Asian Pac J Cancer Prev* 2012, **13:**5887-5895.

66. Joshi RK, Badakar V: **Chemical composition and in vitro antimicrobial activity of the essential oil of the flowers of Tridax procumbens.** *Nat Prod Commun* 2012, **7:**941-942.

67. Appiah-Opong R, Nyarko AK, Dodoo D, Gyang FN, Koram KA, Ayisi NK: **Antiplasmodial activity of extracts of Tridax procumbens and Phyllanthus amarus in in vitro Plasmodium falciparum culture systems.** *Ghana Med J* 2011, **45:**143-150.

68. Kamaraj C, Bagavan A, Elango G, Zahir AA, Rajakumar G, Marimuthu S, Santhoshkumar T, Rahuman AA: **Larvicidal activity of medicinal plant extracts against Anopheles subpictus & Culex tritaeniorhynchus.** *Indian J Med Res* 2011, **134:**101-106.

69. Martin-Quintal Z, Moo-Puc R, Gonzalez-Salazar F, Chan-Bacab MJ, Torres-Tapia LW, Peraza-Sanchez SR: **In vitro activity of Tridax procumbens against promastigotes of Leishmania mexicana.** *J Ethnopharmacol* 2009, **122:**463-467.

70. Tiwari U, Rastogi B, Singh P, Saraf DK, Vyas SP: **Immunomodulatory effects of aqueous extract of Tridax procumbens in experimental animals.** *J Ethnopharmacol* 2004, **92:**113-119.
